# Iron deficiency in barley plants: phytosiderophore release, iron translocation, and DNA methylation

**DOI:** 10.3389/fpls.2015.00514

**Published:** 2015-07-09

**Authors:** Marika Bocchini, Maria Luce Bartucca, Simona Ciancaleoni, Tanja Mimmo, Stefano Cesco, Youry Pii, Emidio Albertini, Daniele Del Buono

**Affiliations:** ^1^Department of Agricultural, Food and Environmental Sciences, University of PerugiaPerugia, Italy; ^2^Faculty of Science and Technology, Free University of BolzanoBolzano, Italy

**Keywords:** iron deficiency, barley, phytosiderophores release, DNA methylation, MSAP

## Abstract

All living organisms require iron (Fe) to carry out many crucial metabolic pathways. Despite its high concentrations in the geosphere, Fe bio-availability to plant roots can be very scarce. To cope with Fe shortage, plants can activate different strategies. For these reasons, we investigated Fe deficient *Hordeum vulgare* L. plants by monitoring growth, phytosiderophores (PS) release, iron content, and translocation, and DNA methylation, with respect to Fe sufficient ones. Reductions of plant growth, roots to shoots Fe translocation, and increases in PS release were found. Experiments on DNA methylation highlighted significant differences between fully and hemy-methylated sequences in Fe deficient plants, with respect to Fe sufficient plants. Eleven DNA bands differently methylated were found in starved plants. Of these, five sequences showed significant alignment to barley genes encoding for a glucosyltransferase, a putative acyl carrier protein, a peroxidase, a β-glucosidase and a transcription factor containing a Homeodomin. A resupply experiment was carried out on starved barley re-fed at 13 days after sowing (DAS), and it showed that plants did not recover after Fe addition. In fact, Fe absorption and root to shoot translocation capacities were impaired. In addition, resupplied barley showed DNA methylation/demethylation patterns very similar to that of barley grown in Fe deprivation. This last finding is very encouraging because it indicates as these variations/modifications could be transmitted to progenies.

## Introduction

Iron (Fe) represents an essential nutrient for all organisms, due to its fundamental role in numerous cellular processes and functions. Its deficiency is a very serious problem for human nutrition (Hell and Stephan, 2003). For instance, in 1995 it has been documented that Fe deficiency was affecting almost 4 billion people worldwide, whilst in 2002 it has been estimated that almost 30% of human population was anemic (Hell and Stephan, 2003). The occurrence of Fe deficit in humans is mainly caused by the consumption of food with low Fe levels. An inadequate Fe intake from diet can lead to varying degrees of deficiency (Kobayashi and Nishizawa, 2012). Crops are the major source of Fe to humans and animals (Hell and Stephan, 2003).

Despite its usually high abundance in the geosphere, Fe is characterized by a scarce solubility in soils, which restricts its availability to plant roots (Mimmo et al., 2014). In particular, the Fe(III) solubility decreases strongly at increasing pH values of soils. This is the consequence of hydrolysation (as Fe-oxohydroxides), polymerization and precipitation reactions (Neilands et al., 1987). These processes can reduce the levels of Fe in soils below those required for an adequate plant growth. The alteration of Fe content of plants is of great importance, since Fe is one of the most yield and quality limiting crop nutrient in the world (Schachtman et al., 1998).

In order to assimilate enough Fe, plants regulate its absorption in response to its availability in soils (Römheld and Marschner, 1986). In fact, *Strategy I* plants improve Fe uptake, acidifying the rhizosphere, by excreting protons by a plasma membrane H^+^-ATPase (Hell and Stephan, 2003).

Thereafter, Fe is reduced at the root surface to Fe(II), through a ferric-chelate reductase, and taken up by a specific Fe transporter (Hell and Stephan, 2003). Differently, *Strategy II* plants, which comprises grasses and graminaceus species (Hell and Stephan, 2003), base their capacity to take up Fe on the release of phytosiderophores (PSs), which are organic compounds with a strong chelation affinity for Fe(III). The Fe-PS complex is then transported into root cells through a high affinity uptake system (Curie et al., 2001; Inoue et al., 2009). PSs belong to the family of mugineic acids (MA), which are biosynthetized starting from the amino acid methionine and then converted into nicotianamine (NA). NA is one of the most important specific Fe chelators operating in the cells. Successively, the deamination of NA leads to oxyMA, which is then hydroxylated to MA (Takahashi et al., 1999). The concentration and kinds of PSs released by plants into the rhizosphere differ between plant species (Mori, 1999). In barley PSs are released with a diurnal trend, showing a peak in the morning (Takagi et al., 1984). Once entered into the cells, Fe is compartmentalized for its uses, but also to avoid an excessive accumulation, which can even lead to cytotoxicity. The highest concentration of Fe is in chloroplasts, for photosynthetic purposes, and in mitochondria in order to carry out the cellular respiration (Mimmo et al., 2014).

Generally, plants modulate gene expression either to cope with environmental changes or in order to counteract biotic and abiotic stresses. The induction of Fe acquisition-related genes under Fe shortage is particularly significant for both *Strategy I* and *II* plants (Hell and Stephan, 2003). Certainly, the purpose of these metabolic regulatory mechanisms is to achieve an adequate supply of this nutrient. Nonetheless, also a number of unexpected proteins and genes, whose exact role is still unknown, respond under Fe deficiency. For example, genes encoding factors that sense intracellular levels of Fe, transcriptional activators for regulating gene expression in response to Fe-deficiency, and components of signaling pathways to monitor Fe status in the environment, have not yet been identified (Negishi et al., 2002).

Although environmental conditions like soil, light, temperature, and microbial activity have been reported to influence Fe uptake and storage (Kokot and Phuong, 1999; Lueders and Friedrich, 2000; Vansuyt et al., 2000), genetic regulatory factors, such as DNA methylation and co-suppression may also play a role (Finnegan et al., 2000; Meins, 2000). In this regard, cytosine methylation may play an integral role in the regulation of gene expression at both the transcriptional and post-transcriptional levels. Specifically, DNA methylation results in the conversion of the cytosine to N4- or N5-methylcytosine or of the adenine to N6-methyladenine. Changes on the methylation status of these cytosine residues in genomic DNA play a pivotal role in the regulation of genome functions (Causevic et al., 2005). Generally, hypermethylation is correlated with gene silencing, while hypomethylation is connected with active transcription (Paszkowski and Whitham, 2001). In addition, Zhang et al. (2006) suggest that body-methylated genes are constitutively expressed at a higher level, whereas promoter-methylated genes tend to be expressed in a tissue-specific manner. DNA methylation is very sensitive to different stresses and indicate how plants adapt themselves to cope with these situations. Several previous researches have demonstrated that cytosine methylation categorically plays important role in regulating various biotic and abiotic stresses such as low temperature (Steward et al., 2000), water deficiency (Labra et al., 2002), bacteria blight (Sha et al., 2005), ion implantation (Yu et al., 2011), hybridization (Hegarty et al., 2011), heavy metals (Aina et al., 2004), salt stress (Marconi et al., 2013), low nutrients (Kou et al., 2011), and tissue culture (Gao et al., 2010; Dann and Wilson, 2011). Nevertheless, scanty genome-wide DNA methylation information is available for *Hordeum vulgare* L. Barley DNA methylation was investigated by Shan et al. (2012) and Smith et al. (2014), but to the best of our knowledge, there is no information regarding barley methylation related to mineral nutrient deficiency/stress. Therefore, a study on methylation patterns in such an important crop as *H. vulgare* under Fe deficiency stress becomes relevant to support knowledge about general genome methylation in this species and regarding the interaction between change in DNA methylation and Fe stress.

On the basis of these assumptions, in the present study we investigated Fe deficient and sufficient barley plants. We chose this species since it is an important crop, and a *Strategy II* plant, which releases PS under Fe shortage. We investigated the effect of Fe deprivation on plant growth, chlorophyll content, on the concentrations of PS released by roots, the Fe content in shoots and roots and the changes in the DNA methylation status caused by the Fe deprivation. To better evidence the plant responses, the above parameters were also assessed in Fe deficient barley plants resupplied with Fe.

## Materials and methods

### Plant material and growth conditions

Barley (*Hordeum vulgare* L. research line Europa) seeds were placed in Petri dishes and added of ultrapure water. Four days later, the seedlings were positioned and grown in continuously aerated hydroponic solutions (12/12 h of light/dark, 23/19°C) composed as follows: 2 mM Ca(NO_3_)_2_ × 4H_2_O, 0.5 mM MgSO_4_ × 7H_2_O, 0.7 mM K_2_SO_4_, 0.1 mM KCl, 0.1 mM KH_2_PO_4_, 1 μM H_3_BO_3_, 0.5 μM MnSO_4_ × H_2_O, 0.5 μM CuSO_4_, 0.5 μM ZnSO_4_ × 7H_2_O, 0.01 μM and (NH_4_)_6_Mo_7_O_24_ × 4H_2_O and ± 100 μM Fe-EDTA to carry out experiments ±Fe deprivation. The nutrient solutions, which had a pH of 6.0 independently to the presence of Fe-EDTA, were renewed every 4 days.

Shoots and roots length together with the chlorophyll concentration (SPAD measurements—SPAD-502 Plus, Konica Minolta, Japan), were assessed on seedlings starting from 9 days after sowing (DAS). SPAD measurements were taken on the first leaf of each plant, 5–10 cm from the bottom, midway between the midrib and the leaf margin. The measurements were then transformed into chlorophyll content (Markwell et al., 1995).

### Resupply experiments

Barley plants were grown in Fe deficiency as described above. At 13 DAS, a subset of Fe deficient plants were subjected to Fe resupply by adding Fe-EDTA (100 μM) to the nutrient solution. Plants were harvested at 2, 4, and 6 days after this treatment for subsequent analyses. Studies on the DNA methylation status were carried out on plants harvested at 2 and 6 days after Fe resupply.

### Root exudates collection and PS quantification

Barley plants were picked up at different times after sowing and submitted to the procedure for the determination of the amount of PS released (Shenker et al., 1995). Briefly, root exudates were collected from plants in the morning, 2 h after the beginning of the photoperiod. After accurate washing of the roots, 3 plants per sample were placed into beakers containing 20 mL of ultrapure water. Roots exudates were collected for 5 h under continuous aeration. The concentration of PS exuded was then quantified colorimetrically using the Cu-CAS assay (Shenker et al., 1995).

### Determination of shoot and root Fe concentration

Barley plants were harvested and then roots and shoots were separated, weighed, and oven-dried at 60°C until constant weight was reached. Plant tissues were microwave digested with concentrated nitric acid (65% v/v, Carlo Erba) using a single reaction chamber (SRC, Ultra WAVE, Milestone Inc, Shelton, CT, USA). Fe concentration was then determined by Inductively Coupled Plasma—Optical Emission Spectroscopy (ICP-OES, SpectroCirosCCD, Spectro, Germany).

### DNA extraction and methylation sensitive amplified polymorphism (MSAP) analysis

The MSAP protocol was applied according to Marconi et al. (2013). Briefly, genomic DNA was extracted from three Fe-sufficient and three Fe-deficient barley samples, collected at 9, 13, 15, and 19 DAS, as well as from three Fe resupplied samples collected at 2 and 6 days after the nutrient addition, (here after named as 15R and 19R) using the DNeasy Plant Mini Kit (Qiagen). For each sample, 300 ng of the genomic DNA was incubated for 4 h at 37°C in a 45 μl mix containing 5 units *Eco*RI, and 5 units *Hpa*II, 1X Restriction-Ligation buffer (1X NEB Buffer 4, New England Biolabs, added with 0.1 M DTT and 250 ng BSA), 50 pmol *Hpa*II adapter, 50 pmol *Eco*RI adapter, 10 mmol ATP, and 1 unit T4 DNA Ligase. The reaction was stopped by incubation at 65°C for 10 min and then diluted 10 times in 0.1X TE (1 mMTris–HCl, 0.1 mM EDTA, pH 8). The second digestion/ligation reaction was carried out in the same way, except that *Msp*I was used instead of *Hpa*II. Two consecutive PCRs were performed to selectively amplify the *Eco*RI-*Hpa*II and *Eco*RI-*Msp*I fragments. The pre-selective amplification was performed using 5 μl of the above-mentioned diluted mixture, which was added to a 45 μl mix.

Selective amplifications of the diluted pre-selective amplified products was carried out. For each reaction, 5 μl of 1:10 diluted pre-selective amplified samples was added to 15 μl selective amplification mix, using a total of 8 primer combinations (Table S1) in a final volume of 20 μl, using the same temperature profile used for the pre-selection step. One μl of each amplified sample was added to 10 μl of formamide and to 0.3 μl of size standard (Genescan LIZ 500, Life technologies). After denaturation (94°C for 5 min) amplified fragments were separated with the ABI 3130xl Genetic Analyzer (Life Technologies).

As described in Marconi et al. (2013) amplified fragments were divided into four types based on the presence or absence of bands, which resulted from the differential sensitivity of the fragments to digestion by *Msp*I and *Hpa*II.

### Silver staining and DNA sequences of Fe-stress-related fragments

Some samples, which were chosen on the basis of interesting polymorphisms, were run on acrylamide gels and silver stained with the aim of isolating and sequencing the selected bands. Following Marconi et al. (2013), 2.5 μL of selected samples were added to 1X formamide dye and denatured. After denaturation, samples were loaded onto a 5% denaturing polyacrylamide gel, and run for 4-4:30 h at 55 W at 38°C. Gels were then silver stained. The gels were fixed in 10% acetic acid, washed three times with ultrapure water for 2 min, transferred to a silver solution (1.5 g/L AgNO_3_, 0.056% formaldehyde) for 30 min, and then rinsed 1 time with ultrapure water. Image development was carried out with agitation for 8–10 min in developer solution. To stop the development and to fix the gel, 10% acetic acid was added directly to the developing solution and incubated with shaking for 3–5 min. The gel was dried at room temperature.

A total of 16 interesting polymorphic bands were excised from gels, rehydrated with 200 μL of ultrapure water o/n at 4°C. Tubes were centrifuged at 10,000 g for 10 min, and the supernatant transferred into a fresh tube. Aliquots of 6 μl were used as template for re-amplification by PCR in a 16 μL reaction volume using Type-it Microsatellite PCR Kit (Qiagen). All PCR reactions were carried out with the same primer combinations used in pre-selective amplification step with the following profile: 94°C for 1 min, 30 cycles of denaturation at 94°C for 1 min, annealing at 50°C for 1 min, and extension at 72°C for 1 min, ending with a 20 min extension step at 72°C.

One μl of the re-amplified DNA was cloned into a pCR4-TOPO vector using the TOPO TA cloning kit for sequencing (Invitrogen). Three plasmid DNAs for each transformation were purified from 5 ml of overnight cultures of *Escherichia coli* in LB medium using the GenElute Plasmid miniprep kit (Sigma). The sequences of both strands of each plasmid were determined after running sequencing reactions (obtained with BigDye Terminator v3.1 Cycle Sequencing Kit, Life Techologies) on an ABI 3130xl Genetic Analyzer sequencer.

### Statistical analysis

Each reported value represents the mean ± standard deviation (SD) of data from four independent experiments on at least three biological replicates per experiment. Shoot and root length and plant weights were assessed using 20 replicates. Statistical analyses of data were carried out by ANOVA tests and significant differences were established by Duncan's tests at *P* < 0.05. Finally, chi-square was used to test the independence between methylation level and Fe deprivation (stress) condition, using SAS Version 9.2 (SAS Institute, Cary, NC). Student's *t*-test was also performed using SAS software.

## Results

### Length and weight of shoots and roots, and chlorophyll content in barley plants ±Fe

Iron shortage affected the growth of seedlings of barley starting from 15 DAS. In fact, significant reductions in both shoots and roots length and weight were observed (Table 1). In particular, at 15 and 19 DAS, the shoot length of the Fe deficient barley plants was reduced by 12.0 and 20.7%, respectively, with respect to the Fe sufficient samples. Also shoot fresh weight (FW) was significantly affected by Fe shortage. In fact, reductions in shoot FW of 22.2 and 36.0% were found at 15 and 19 DAS, respectively. A similar negative effect occurred to the root length that was reduced by 17.2%, at 19 DAS. Finally, the nutrient privation also caused a 14.2% reduction in the root FW at 19 days after the beginning of the Fe shortage treatment.

**Table 1:** **Length and fresh weight (FW) of shoots and roots of barley plants grown under Fe sufficiency (+Fe) and Fe-deficiency conditions (−Fe), at 9, 12, 13, 15, and 19 DAS**.

**DAS (days after sowing)**					
	**9**	**12**	**13**	**15**	**19**
**SHOOT LENGHT (CM)**					
+Fe	15.30 ± 1.36a	18.63 ± 1.32a	20.20 ± 1.74a	24.87 ± 1.70a	32.07 ± 2.24a
−Fe	14.47 ± 1.52a	17.43 ± 1.50a	18.75 ± 1.57a	21.87 ± 1.38b	25.42 ± 2.06b
**SHOOT FW (G)**					
+Fe	0.18 ± 0.03a	0.25 ± 0.04a	0.24 ± 0.03a	0.27 ± 0.03a	0.39 ± 0.08a
−Fe	0.16 ± 0.04a	0.19 ± 0.06a	0.21 ± 0.02a	0.21 ± 0.03b	0.25 ± 0.03b
**ROOT LENGHT (CM)**					
+Fe	13.00 ± 1.41a	17.30 ± 2.84a	18.65 ± 3.16a	19.07 ± 2.18a	24.13 ± 2.29a
−Fe	11.30 ± 2.16a	14.57 ± 2.68a	15.55 ± 2.64a	17.67 ± 2.17a	19.97 ± 2.41b
**ROOT FW (G)**					
+Fe	0.11 ± 0.02a	0.12 ± 0.01a	0.11 ± 0.02a	0.12 ± 0.01a	0.14 ± 0.01a
−Fe	0.09 ± 0.01a	0.11 ± 0.01a	0.11 ± 0.01a	0.12 ± 0.01a	0.12 ± 0.01b

Data are means ± SD (n ≤ 20). For each column, means followed by different letters are significantly different at P = 0.05.

**Figure 1:**
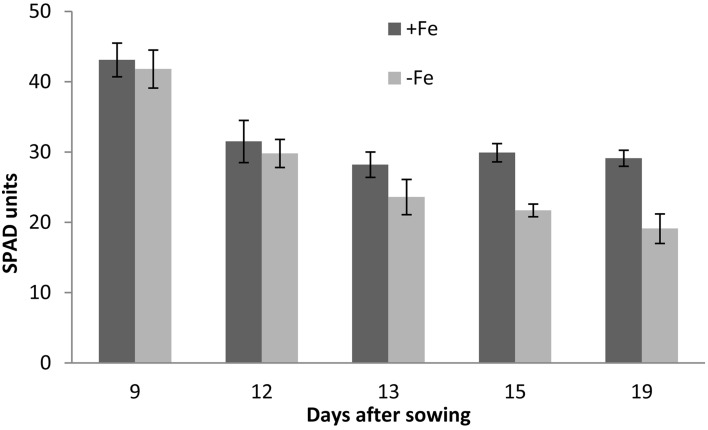
**Chlorophyll concentration in barley shoots grown under Fe sufficiency (+Fe) and Fe-deficiency (−Fe) conditions, at 9, 12, 13, 15, and 19 DAS**. At 9 DAS the SPAD was measured at the first leaves, thereafter, it was recordered on the second leaves.

Changes in chlorophyll contents in response to the Fe starvation were assessed in Fe deficient leaves of barley by a SPAD meter (Figure 1). The leaf chlorophyll level was significantly reduced in the Fe deficient plants at 13, 15, and 19 days after this nutrient deprivation and the reductions were of 16.3, 27.4, and 34.5%, respectively.

### Phytosiderophores release

**Figure 2:**
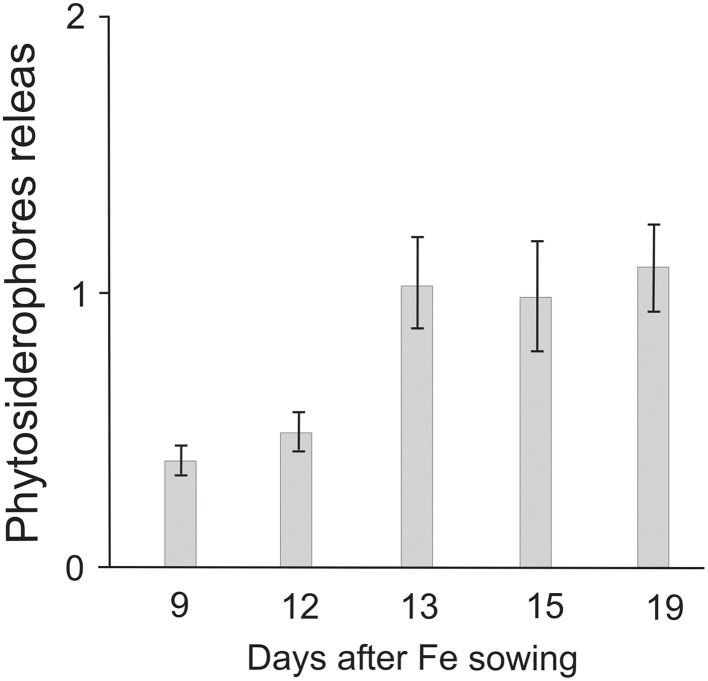
**Phytosiderophores release (μmol g^−1^ RFW) from barley plants grown under Fe-deficiency at 9, 12, 13, 15, and 19 DAS**.

Root exudates were collected from barley plants grown under Fe deficiency in hydroponic solutions. The quantification of exudates was started 8 DAS, but, at this time, the concentration of these organic compounds was scarce and far below the limit of detection of the method (30 μM). Starting from 9 DAS, Fe deficient plants began to exude higher concentrations of PSs, and this exudation pattern was found to be quite constant until 12 DAS (Figure 2). On the other hand, starting from 13 DAS the amount of extruded PSs by the plants strongly increased. In particular, at 13, 15, and 19 DAS Fe deficient barley exuded 1.03, 1.00, and 1.10 μmol g^−1^ RFW, respectively.

### Fe concentration in barley plants

Iron concentration was determined in both shoots and roots of barley plants grown in either a complete or Fe-free hydroponic nutrient solution (Table 2). As expected, Fe deprivation strongly affected the Fe content in barley and its distribution between shoots and roots (Table 2). The differences between Fe content deficient and sufficient plants were found to be significant already at 8 DAS. In particular, Fe sufficient barley showed a total Fe content (shoots + roots) of 19.49 μg g^−1^ FW, while Fe deficient barley samples evidenced a total Fe content of 14.37 μg g^−1^ FW. The Fe root to shoot ratio was found to be significantly higher in Fe fed barley and reached values of 2.15, 3.55, 9.48, 9.70, and 16.92, at 8, 9, 13, 15, and 19 DAS, respectively. This ratio was found to be decreased in Fe deficient barley plants already at 8 DAS (Table 2), if compared to Fe sufficient plants, and reached the values of 1.13, 1.08, 1.09, 1.61, and 2.00 at 9, 13, 15, and 19 DAS, respectively (Table 2).

**Table 2:** **Iron concentration in shoots and roots of barley plantsgrown in iron sufficiency (+Fe) and iron deficiency (−Fe) conditions, at 8, 13, 15, and 19 DAS**.

**DAS**	**[Fe]_shoots_(μg g^−1^ FW)**		**[Fe]_roots_(μg g^−1^ FW)**		**[Fe]_roots/shoots_ (ratio)**	
	**+Fe**	**−Fe**	**+Fe**	**−Fe**	**+Fe**	**−Fe**
8	6.18 ± 0.43a	6.45 ± 0.65a	13.31 ± 1.23a	7.92 ± 1.64b	2.15	1.13
9	6.05 ± 0.33a	5.24 ± 0.48a	21.52 ± 2.67a	5.65 ± 1.18b	3.55	1.08
13	6.19 ± 0.62a	4.28 ± 0.52b	58.71 ± 5.55a	4.68 ± 0.25b	9.48	1.09
15	5.79 ± 1.39a	3.33 ± 0.32b	56.18 ± 2.55a	5.37 ± 0.50b	9.70	1.61
19	5.50 ± 1.125a	2.81 ± 0.50b	93.07 ± 4.30a	5.46 ± 0.28b	16.92	2.00

Data are means ± SD (n = 20). Means within the same row followed by the same letter are not significantly different from the relative +Fe controls.

### Chlorophyll and Fe concentrations in barley shoots and roots after Fe resupply

**Figure 3:**
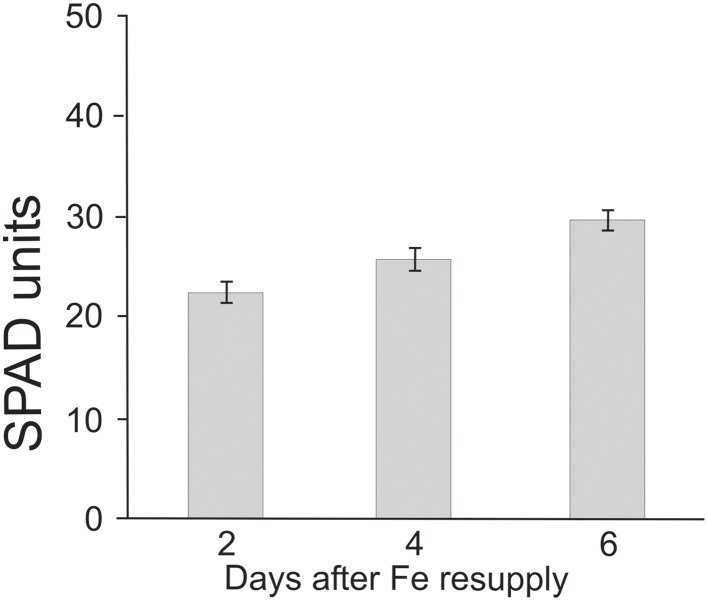
**Chlorophyll concentrations in barley shoots grown in Fe-deficiency conditions (-Fe), resupplied with Fe-EDTA 100 μM at 13 DAS**.

Iron deficient barley plants were resupplied with 100 μM Fe-EDTA at 13 DAS. The monitoring of chlorophylls evidenced that leaves of Fe-resupplied plants reached the SPAD values recorded in control plants over a period of 6 days (Figure 3). In particular, the chlorophyll recovery became evident at 4 days after the resupply (Figure 3).

Regarding the Fe concentrations, the resupply experiments (Table 3) evidenced, at 2 days after this nutrient addition, some increases in the Fe root to shoot ratio (2.59). The recovery of Fe resupplied plants continued until 6 days after this nutrient addition, when the root to shoot ratio reached the value of 8.87 (Table 3). Nonetheless, these samples did not reach the values exhibited by Fe sufficient controls (Table 2).

**Table 3:** **Iron concentration in shoots and roots of barley plants grown in iron deficiency (−Fe) conditions, resupplied with 100 μM Fe-EDTA at 13 DAS**.

**Days after resupply**	**[Fe]_shoots_ (μg g^−1^ FW)**	**[Fe]_roots_ (μg g^−1^ FW)**	**[Fe]_roots/shoots (ratio)_**
2	3.41 ± 0.16	8.82 ± 0.15	2.59
4	3.49 ± 0.13	23.73 ± 3.25	6.80
6	3.88 ± 0.19	34.42 ± 2.74	8.87

Data are means ± SD (n = 3).

### Extent and pattern of DNA methylation under control conditions and Fe deficiency

Eight primer combinations (Table S1) were used to assay cytosine methylation at 5'-CCGG-3′ sequences in the Europa research line of *H. vulgare*, at different times after sowing, in Fe deficient/sufficient plants. Samples were collected from three plants grown ±Fe at 7, 9, 13, 15, and 19 DAS. In addition, for the MSAP analysis, other samples were collected at 2 and 6 days after Fe-EDTA100 μM resupply (hereafter named 15R and 19R). 7 DAS samples were collected before treatment and therefore there are not stressed samples. They represented our T0.

A total of 563 clear and reproducible bands were amplified from plants growth ±Fe. Under the 7, 9, 13, 15, and 19 DAS control conditions, the total methylation of CCGG sequences averaged 61.28, 62.34, 60.92, 62.17, and 62.88% respectively, while the extent of DNA methylation ranged from 59.33 (9 DAS) to 61.99% (13 DAS) in stressed samples (Table 4). In particular, when compared with the respective Fe well-fed controls, DNA methylation levels in Fe deficient samples decreased (−3.02% in 9 DAS, −2.66% in 15 DAS and −1.24% in 19 DAS); the only exception was at 13 DAS when an increase of 1.07% in DNA methylation was observed.

**Table 4:** **DNA methylation changes at 9, 13, 15, and 19 DAS of barley under Fe deprivation**.

**MSAP band type**	**Control**					**Fe deprived**				**Fe resupplied**	
	**7**	**9**	**13**	**15**	**19**	**9**	**13**	**15**	**19**	**15R**	**19R**
I	218	212	220	213	209	229	214	228	216	223	225
II	62	71	71	77	65	72	80	69	82	79	76
III	209	210	228	223	234	222	233	224	232	224	219
IV	74	70	44	50	55	40	36	42	33	37	43
Tot. Amplified bands	563	563	563	563	563	563	563	563	563	563	563
Tot. methylated bands^a^	345	351	343	350	354	334	349	335	347	340	338
Fully methylated bands^b^	283	280	272	273	289	262	269	266	265	261	262
Hemi-methylated bands^c^	62	71	71	77	65	72	80	69	82	79	76
MSAP (%)^d^	61.3	62.3	60.9	62.2	62.9	59.3	62.0	59.5	61.6	60.4	60.0
Fully methylated ratio (%)^e^	50.3	49.7	48.3	48.5	51.33	46.5	47.8	47.2	47.1	46.4	46.5
Hemi-methylated ratio (%)^f^	11.0	12.6	12.6	13.7	11.5	12.8	14.2	12.3	14.6	14.0	13.5

a (II+III+IV).

b (III+IV).

c (II).

d MSAP (%) = [(II+III+IV)/(I+II+III+IV)] × 100.

e Fully methylated ratio (%) = [(III+IV)/(I+II+III+IV)] × 100.

f Hemi-methylated bands (%) = [(II)/(I+II+III+IV)] × 100.

Type I indicated absence of methylation due to the presence of bands in both EcoRI/HpaII and EcoRI/MspI digest; type II bands appeared only in EcoRI/HpaII digestion but not in the EcoRI/MspI digest; type III generated bands obtained in EcoRI/MspI digest but not in the EcoRI/HpaII digest; and type IV represents the absence of band in both enzyme combinations.

In addition, all samples showed a level of full methylation higher of hemi-methylation (Table 4). Fe deficiency caused a general decrease of fully methylated bands (−3.20, −0.53, −1.24, and −4.26% at 9, 13, 15, and 19 DAS, respectively) with respect to control samples. In contrast the level of hemy-methylated amplicons in Fe-deficient samples increased (+0.18, +1.6, and +3.02%) at 9, 13, and 19 DAS, respectively when compared with Fe sufficient samples. The only exception was at 15 DAS when a decrease of 1.42% was observed.

The average level of DNA methylation (60.2%) after the Fe resupply was comparable to that of samples grown under Fe deficiency. The relative abundances of fully methylated bands did not change during the recovery, if compared with -Fe sufficient samples (average of 46.35 and 46.53% in 15R and 19R vs. 47.25% in 15S and 47.07% in 19S).

### Effect of Fe deficiency on the level of methylation in barley

Consistently with the approach used by Marconi et al. (2013), all possible banding patterns between control and Fe deprived barley plants at 9, 13, 15, and 19 DAS were compared for identifying the changes in cytosine methylation patterns under Fe shortage. Sixteen banding patterns were apparent from the MSAP analysis (Table 5). Patterns A–D represent monomorphic classes in which the methylation pattern is the same following either the control or the Fe-deprived samples. Patterns E–J are indicative of cytosine demethylation, whereas possible cytosine methylation events induced by Fe deficiency are represented by patterns K–P.

**Table 5:** **Analysis of DNA methylation patterns in barley plants under Fe deprivation compared with plants well-fed with iron**.

**Pattern^a^**	**Class**	**H_2_O**		**Fe-/Resup**		**Fe deprived**				**Fe resupplied**	
		***Hpa*II**	***Msp*I**	***Hpa*II**	***Msp*I**	**9**	**13**	**15**	**19**	**15R**	**19R**
No change	A	1	0	1	0	52	63	56	62	60	55
	B	0	1	0	1	187	217	207	217	210	209
	C	1	1	1	1	196	205	207	197	205	198
	D	0	0	0	0	27	25	23	30	19	29
	Total					462 (82.1%)	510 (90.6%)	493 (87.6%)	506 (89.9%)	494 (87.7%)	491 (87.2%)
Demethylation	E	1	0	1	1	10	0	4	2	3	6
	F	0	1	1	1	19	8	12	15	9	16
	G	0	0	1	1	4	1	5	2	6	5
	H	0	1	1	0	2	0	0	1	0	1
	I	0	0	1	0	13	15	12	18	19	19
	J	0	0	0	1	26	3	10	5	6	2
	Total					74 (13.1%)	27 (4.8%)	43 (7.6%)	43 (7.6%)	43 (7.6%)	49 (8.7%)
Methylation	K	1	1	1	0	5	2	1	1	0	1
	L	1	1	0	1	8	12	5	10	8	8
	M	1	1	0	0	3	1	0	1	0	2
	N	1	0	0	1	1	1	2	0	0	0
	O	1	0	0	0	8	7	15	1	14	4
	P	0	1	0	0	2	3	4	1	4	8
	Total					27 (4.8%)	26 (4.6%)	27 (4.8%)	14 (2.5%)	26 (4.6%)	23 (4.1%)

a 1, band present in all stages; 0, band absent in all stages.

Methylation at 462 (82.06% of total sites), 510 (90.59% of total sites), 493 (87.57% of total sites), and 506 (89.88% of total sites) CCGG sites remained unchanged after the imposition of Fe deficiency at 9, 13, 15, and 19 DAS, respectively (Table 5).

Under Fe shortage, demethylation was observed for 74 (13.1%) 27 (4.8%), 43 (7.6%), and 43 (7.6%) CCGG sites at 9, 13, 15, and 19 DAS, respectively (Table 5), highlighting a higher level of DNA demethylation soon after the Fe deprivation (Table 5). Methylation due to Fe shortage was generally less abundant accounting for 27 (4.8%), 26 (4.62%), 27 (2.49%), and 14 (4.8%) at 9, 13, 15, and 19 DAS, respectively (Table 5). Therefore, Fe deficiency caused more DNA demethylation rather than DNA methylation (Table 5).

### Alteration of DNA methylation pattern under Fe deprivation and after subsequent resupply

To identify the DNA methylation changes (i.e., demethylation or methylation under Fe deprivation and subsequent resupply), we classified all differentially methylated DNA fragments into various classes. As indicated in Table 6, the a, b, and c classes include bands with DNA demethylation induced by Fe deficiency; the d, e, and f classes comprise methylated DNA fragments induced by Fe shortage; and the g and h classes included DNA fragments for which Fe stress had no effect on their methylation status. The majority of bands (83.65 and 84.66%) remained unchanged (class h) in the Fe resupplied barley plants at 2 (15R) and 6 (19R) days after the Fe addition. As many as 24 (61.54%) and 27 (69.23%) out of 39 demethylated DNA bands remained hypomethylated (class b), whereas 13 (33.3%) and 12 (30.77%) out of 39 demethylated DNA bands were re-methylated (class a) after resupply, at 2 (15R) and 6 (19R) days after the Fe addition, respectively (Table 6). Only few bands (2 and 0at 15R and 19R samples) were found to belong to class c (demethylated by Fe deprivation but re-methylated with a different pattern after resupply).

**Table 6:** **Summary of the changes in the DNA methylation patterns in barley after 2 (15R) and 6 (19R) days of resupply**.

Band class^*^	**a**	**b**	**C**	**a+b+c**	**d**	**e**	**f**	**d+e+f**	**g**	**h**	**i**	**Total**
Recovery 15R	13	24	2	39	9	17	1	27	22	471	4	563
Recovery 19R	12	27	0	39	4	8	1	13	31	475	5	563

* (a) demethylated by Fe deprivation, but remethylated after recovery; (b) demethylated by Fe deprivation, and remaining hypomethylated after recovery; (c) demethylatd by Fe deprivation but re-methylatd in a different pattern after recovery; (d) methylated by Fe deprivation, but demethylated after recovery; (e) methylated by Fe deprivation, and remaining methylated after recovery; (f) methylatd by Fe deprivation, but demethylated in a different pattern after recovery; (g) DNA methylation pattern remained unchanged under Fe deprivation, but changed after recovery; (h) DNA methylation pattern was unchanged under Fe deprivation, and remained unchanged after recovery; (i) others.

A similar behavior was observed in 15R and 19R samples in terms of the methylated DNA fragments induced by Fe deficiency (Table 6). For both samples, resupply affected the methylation status of few of the fragments subject to Fe-induced DNA methylation. In fact, only 9 (33.33%) and 4 (30.77%) out of 27 and 13 bands were found to belong to d class (i.e., methylated by Fe deprivation, but demethylated after resupply) at 15R and 19R, whereas the vast majority of them remained unchanged after resupply from Fe deprivation (62.96% and 61.53%, class e). Only one band of class f (methylated by Fe deprivation and demethylated with a different pattern after resupply) for was observed for both resupplied samples (15R and 19R).

### Sequencing and bioinformatics analysis of methylated DNA fragments

Sixteen differentially methylated DNA bands were cloned and sequenced. The resulting sequences were blasted against the databases at NCBI, IPK, Gramene and Uniprot websites. Five fragments' sequences were too short (between 40 and 70 bp) and resulted in no similarities. As shown in Table 7, the remaining 11 sequences (82–340 bp, with an average of 220 bp) scored when aligned to plant databases.

**Table 7:** **Functional association of the methylated fragments**.

**MSAP band**	**Size (bp)**	**Chr. Location**	**Accession number, putative function, and Blast score**
Hv_01	290		I2GL33^*^; oxidoreductase domain protein[*Fibrisoma limi* gen. nov., sp. nov.]; 3.5e-36
Hv_02	118	4	AT4G30660; proteolipid membrane potential modulator [*Arabidopsis thaliana* L.]; 7.5
Hv_03	120	7	AK373414; glucosyltransferase [*Hordeum vulgare* L.], 0.004
Hv_04	300		AK364780; cysteine protease family protein putative [*Hordeum vulgare* L.]; 1.4
Hv_05	115		AK364906; unknow; [*Hordeum vulgare* L.]
Hv_06	340	2	MOX2R2^*^ acyl_carrier_prot-like; [*Hordeum vulgare* L.]; 1.9e-164
Hv_07	290		AK375528; peroxidase-like superfamily [*Hordeum vulgare* L.]; 3e-34
Hv_08	82		AAV80394; nucleic acid binding; [*Hordeum vulgare* L.]; 7.9
Hv_09	83		EF067844.1 *vrs1 locus and Hox1* gene; [*Hordeum vulgare* L.]; 0.003
Hv_10	259		AK370298; Uncharacterized protein- GHMP-kinase C terminal domain [*Hordeum vulgare* L.]; 8.9
Hv_11	260		AK250128; beta-glucosydase [*Hordeum vulgare* L.]; 5e-11

Resulting sequences were blasted against the databases at NCBI, IPK, Gramene, and Uniprot websites.

* UniProt ID.

Six sequences aligned well with at least one plant database sequence (*e*-value lower than 0.05) (i.e., Hv_01, Hv_03, Hv_06, Hv_07, Hv_09, and Hv_11); among these, five sequences were significantly associated with *H. vulgare* genes; (i) one (accession number AK373414) encodes for a glucosyltransferase; (ii) one (Uniprot accession number MOX2R2) encodes for an acyl carrier protein; (iii) a third one (accession number AK375528) gives a peroxidase protein; (iv) one (accession number EF067844.1) encodes a Homeobox-leucine zipper protein *HOX1*; and (v) the fifth sequence score well with a β-glucosydase protein (accession number AK250128). It is worth noting that also the other 4 sequences resulted in similarities with either barley or Arabidopsis genes even if with no significant *e*-values.

## Discussion

Iron deficiency is a widespread problem affecting more and more cropping systems and thus causing negative impacts on crop productions. Specific soil characteristics are the main cause for low Fe availability to plant roots (Römheld and Marschner, 1986); for instance, one third of the cultivated areas is considered Fe-deficient (Mori, 1999). For these reasons, this study was aimed at investigating in barley, on a wide temporal scale, the effects of Fe deficiency on growth, phytosiderophores (PSs) release, Fe content and on methylation status of DNA. To the best of our knowledge, there is no literature on barley describing the effects of Fe deficiency on DNA methylation status.

Results of our experiments indicate that, as expected, shoot and root biomass production was significantly reduced by Fe starvation (Table 1). In particular, shoot length and weight were affected at 15 DAS, while root length and weight were decreased at 19 DAS. The magnitude of these negative effects was found to be of more consistent entities on the aerial biomass (Table 1). Van der Werf and Nagel (1996) documented that plants grown in sufficient availability of nutrients invest more energy in constructing aerial biomass than roots. *Viceversa*, in situations of low nutrient availability, plants respond to this stress by reducing preferably aerial biomass than roots (Van der Werf and Nagel, 1996).

Regarding Fe content, we found that plant ±Fe showed significant differences in this nutrient content already at 8 DAS. At this date, it can be reasonably assumed that seeds Fe content was already exhausted (Yousfi et al., 2009). Consequently, the early chlorosis symptoms were now depending on the rate of leaves expansion (Yousfi et al., 2009). In addition, Fe deficient barley seemed to slightly decrease the rate of Fe translocation to leaves. As indicated in Table 2, the root to shoot ratios varied from 1.13 at 8 DAS to 2.00 at 19 DAS. Fe redistributions were observed also in other plants, and this trait is considered an aptitude of plants to guarantee an adequate Fe transport to their leaves (Yousfi et al., 2009).

Chlorophylls concentrations, expressed as SPAD index, was strongly reduced by Fe starvation (Figure 1). In fact, decreases were continuous and progressive from 13 to 19 DAS (Figure 1). The establishment of chlorosis was found to be quite successive to the alteration in the Fe content in starved barley (Table 2). This asynchrony is due to the chlorophylls dilution in leaves growing at normal rates in Fe deficiency (Abadía et al., 2000).

The release of PS is known to be depending on the Fe content of plants (Mimmo et al., 2014). In fact, the presence/absence of Fe in tissues deactivate/activate biosynthesis and release of these organic ligands (Mimmo et al., 2014). Our experiments showed that plants started to release PSs at 9 DAS. The concentration of exuded ligands more than doubled at 13 DAS (Figure 2). In addition, data of Table 2 and Figure 2 indicate that PS release started at 9 DAS, when the Fe concentration in the roots of Fe deficient plants was found significantly lower than that of Fe sufficient roots (Table 2). This result indicates a correlation between the roots Fe contents and the beginning of PS exudation. On the other hand, shoots of Fe deficient plants started 13 DAS to show lower Fe concentrations than well fed ones. At this time, the PSs release resulted more than doubled (Figure 2). These findings would be discussed by considering that the control of the Fe status in plants is regulated by sensors which are localized both in leaves and roots (Schmidt, 2003). In particular, the sensor allocated in shoots would promote the synthesis and translocation to roots, thorough phloem, of signal molecules. Then, plants would biosynthesize and release PSs into rhizosphere (Schmidt, 2003). Differently, the sensor in roots would modulate the signals received by shoots, which is in turn regulated by the Fe content of apoplast (Schmidt, 2003). Our experiments indicate that the low initial amount of exuded PS (from 9 to 12 DAS), was mainly correlated with the alteration in the Fe-content in the roots (Table 2). In fact, Fe deficient barley roots showed at 9 DAS a Fe content much lower than the Fe sufficient plants (5.65 vs. 21.52 μg g^−1^ FW). At 13 DAS, a significant decrease in the Fe content was found in the shoots of the starved plants (4.28 μg g^−1^ FW), with respect to those of Fe sufficient samples (6.19 μg g^−1^ FW). This finding indicates that the massive release of PSs at 13 DAS was mainly correlated to the reduced Fe concentration in the shoots.

Resupply studies conducted after the addition to starved plants 13 days old of Fe-EDTA 100 μM, evidenced some interesting trends. In particular, the chlorophylls estimation (Figure 3) indicate that this variety of barley reached a normal SPAD index in 6 days after Fe addition. Relatively to Fe contents (Table 3), resupplied plants immediately started to increase Fe concentration at roots level, while the translocation to the aerial parts seemed to be very slow, without achieving, after 6 days of resupply, values comparable to those of unstressed controls. Finally, it is to be mentioned that PS exudation of resupplied plants (data not shown) did not substantially change from that of Fe deficient plants (Figure 2). Just some slight decreases were ascertained at 6 days after Fe addition. This trend seems to confirm that Fe concentration in shoots plays the major role in controlling biosynthesis and exudation of these compounds.

In this paper, the MSAP technique was employed in order to determine the level of DNA methylation in *H. vulgare* and to ascertain its changes in result of Fe deficiency. The overall rate of methylation observed (60%) is higher than that found by Smith et al. (2014) and similar to that found by Shan et al. (2012) in the same species. In addition our results indicate that in barley the level of methylation is higher than that of *Gossypium hirsutum* (37%), *Triticum aestivum* (38%), *Trifolium repens* (28%), and *Cannabis sativa* (23%) (Aina et al., 2004; Zhong et al., 2009; Cao et al., 2011) and similar to that of *Brassica oleracea* (Salmon et al., 2008), *Oryza sativa* L. (Karan et al., 2012), and *Brassica napus* var. *oleifera* (Marconi et al., 2013).

Our data highlighted important differences between the amount of fully and hemy-methylated bands. In particular, in control samples, fully, and hemy-methylated bands represented the 49.6 and 12.3% of the total bands, respectively, while in the Fe deprived samples these ratios were 47.15 and 13.5%, respectively. These results show a small decrease of fully methylated bands and a small increase of hemy-methylated bands in Fe deprived samples, when compared with Fe well-fed ones. These percentages are similar to those seen by Marconi et al. (2013) in a salt tolerant cultivar of rapeseed and, limited to hemy-methylated bands, in cotton (Cao et al., 2011). Lower values were found in rice genotypes under salt stress by Wang et al. (2011) and Karan et al. (2012), in a salt-sensitive cultivar of rapeseed by Marconi et al. (2013), and in both white clover and industrial hemp by Aina et al. (2004). All together, these studies suggests that the amount of fully and hemy-methylated bands depends both on the species, and on differences between genotypes belonging to the same species.

In our samples, we observed a very small variation between stages either for Fe well-fed samples or in Fe-deprived and resupplied samples for each class of fragment (Table 5) and this was also confirmed by a Chi-square Test (data not shown). Our results showed that, although cytosine methylation levels remained unchanged or similar between both control and Fe-deprived staged samples, this level varied when the same stages (DAS) of Fe well-fed samples were compared with Fe-deprived ones (Table 5).

Methylation changes due to Fe deficiency in this experiment occurred very rapidly. In fact (Table 5) the demethylation ratio in samples at 9 DAS (13.1%) was much higher than the methylation one (4.8%). At 19 DAS the amount of cytosine demethylation (7.6%) was alike that recorded for the previous stage, while methylation decreased from 4.8% at 17 DAS to 2.5% at 19 DAS. The level of methylation/demethylation recorded in this study confirm results obtained with the same technique in other plant species subjected to environmental changes (i.e., Arabidopsis, clover, hemp, tobacco, and wheat; Lízal and Relichová, 2001; Aina et al., 2004; Choi and Sano, 2007; Zhong et al., 2009). In addition, the results reported in this study suggests that Fe-deprivation induced more DNA demethylation than DNA methylation; these results are consistent with previous studies that showed that abiotic stresses tends to demethylate genomic DNA (Aina et al., 2004; Choi and Sano, 2007; Zhong et al., 2009; Cao et al., 2011; Wang et al., 2011).

Finally, a large amount cytosine methylated/demethylated during Fe-deprivation remained methylated/demethylated after resupply (i.e., 77% at 15R and 61.5% at 19R and 61% at 15R and 69% at 19R, respectively, Table 6). This suggested that variation in methylation/demethylation was maintained even once the deficiency was removed and could likely be transmitted to progeny.

Six nucleotide sequences were significantly associated with barley genes (Table 7). Hv_03 displayed sequence homology with a gene encoding for a glucosyltransferase. These enzymes catalyze glucosylation reactions, by forming a glucosydic bond (Liu and Mushegian, 2003), and are involved in plant stress responses to harmful metabolites and toxic environmental compounds (Edwards et al., 2005). Regarding to Fe starvation, a gene encoding for an UDP-glucosyltransferase protein was found to be up-regulated in *Glycine max* grown in Fe deficiency conditions (O'Rourke et al., 2007).

Hv_06 sequence was very similar a *H. vulgare* putative acyl carrier protein (ACP), which is believed to play a central role in fatty acid biosynthesis in all living organisms, carrying the acyl chains through the various steps in fatty acid biosynthesis (Chan and Vogel, 2010). Recently, it has been shown that in *Chlamydomonas reinhardtii* Fe deficiency causes the formation of intracellular lipid droplets and a quick down-regulation of genes involved in the *de novo* synthesis of fatty acid, among which ACP1/2 encoding for the acyl carrier protein 1 (Urzica et al., 2013). Hv_07 nucleotide sequence showed a significant sequence similarity with a barley gene that encodes for a protein belonging to the peroxidase superfamily, which are ubiquitous enzymes involved in many physiological processes pivotal for plant life (Passardi et al., 2005). Peroxidases are mainly implicated in the cellular regulation of reactive oxygen species (ROS) and hydrogen peroxide (Passardi et al., 2005). Since Fe is a cofactor for many antioxidant enzymes, Fe deficient plants can be expected to be more sensitive to oxidative stress. To date, very few attention has been paid to the question wheatear Fe starvation can impair antioxidant defenses in plants. However, some evidence were reported on the correlation between low Fe availability and reduced peroxidases and superoxide dismutase activities, in sunflower and *A. thaliana* (Ranieri et al., 2001; Ramírez et al., 2013).

The six-rowed spike 1 (vrs1) gene, was the best match for Hv_09 DNA. The six-rowed phenotype is genetically determined by homozygosity for the recessive allele at the *vrs1* locus, which has been identified as a homeobox gene (*HvHox1*) (Sakuma et al., 2009). This gene encodes for a transcription factor containing a homeodomain (HD). HD is a 60 amino acids motif present in a number of eukaryotic transcription factors, frequently involved in developmental processes (Gago et al., 2002). Recently, this gene has been associated to the regulation of plant stress responses correlated with changes in environmental conditions and water deficit (Elhitia and Stasolla, 2009). The sequence of Hv_11 DNA led to the identification of a *H. vulgare* gene encoding for a putative β-glucosidase, an enzyme involved in the mobilization of complex carbohydrates, and this is in line with the observation that Fe deficient plants generally display higher sugar concentrations than Fe sufficient ones (López-Millán et al., 2000). In addition, an extracellular β-glucosidase, isolated from barley leaves, was found to be able to catalyze the de-esterification of glucosyl-abscisic acid to generate ABA (Dietz et al., 2000). It has been proposed that this activity was involved in the modulation of stress signals and gene expression in cells (Dietz et al., 2000). Finally, Zamioudis et al. (2014) showed that a β-glucosidase from *A. thaliana* was responsive to Fe starvation and involved in the secretion of Fe-mobilizing phenolic metabolites, in the close proximity of the roots.

Since agriculture is strictly depending on mineral nutrition, and most of the nutrients are present in the soil in unavailable forms, the knowledge of how plants mobilize them, by releasing organic compounds, is of pivotal importance. Consequently, the understanding of how the plants modulate gene expression to cope with nutrients fluctuations or deficiency appears even more important. Concerning our study, we ascertained that barley plants responded to iron starvation by activating some physiological and biochemical changes, in order to cope with this adverse nutritional situation. In particular, Fe starved barley plants exuded huge amounts of organic ligands in correlation with the aerial and radical Fe contents. Finally, the MSAP technique was employed to ascertain the level of DNA methylation/demethylation of barley genome, and its variations in Fe deficiency. Not only the results demonstrated a clear effects of Fe starvation on the status of fully and hemy-methylated bands, but also the resupply experiments suggested, at least, that the methylation/demethylation status could be maintained even once the Fe deficiency is removed. The relevance of this last finding is supported by the fact that such modifications could likely be transmitted to progeny.

## Author contributions

Conceived and designed the experiments: EA, DD, MB, MLB, YP, TM, SCe. Performed the experiments: MB, MLB, YP, SC. Analyzed the data: EA, DD, MB, MLB, TM, SC, YP. Contributed reagents/materials/analysis tools: EA, DD, SCe. Wrote the paper: EA, DD, MB, TM, SC.

### Conflict of interest statement

The authors declare that the research was conducted in the absence of any commercial or financial relationships that could be construed as a potential conflict of interest.
