# Supplementary material for: Iron deficiency in barley plants: phytosiderophore release, iron translocation, and DNA methylation
Source: Front Plant Sci. 2015 Jul 9;6:514. doi: 10.3389/fpls.2015.00514 (PMC4496560; doi:10.3389/fpls.2015.00514)
Supplement: Supplementary file 1 [file Table1.PDF]

Table S1. MSAP primer combination.

Name	Sequence
ECO RI (E)	5'-GACTGCGTACCAATTC
HPA/MSP (HM)	5'-GATGAGGTCTAGAACGG
**MSAP primer combinations**	
E-CAG/HM-ACT	E-ACA/HM-TGA
E-CCA/HM-AAT	E-ACA/HM-TCG
E-CCA/HM-ATC	E-AGC/HM-TAG
E-CCA/HM-ACT	E-ACA/HM-TAC
